# Increased dosing regimens of piperacillin-tazobactam are needed to avoid subtherapeutic exposure in critically ill patients with augmented renal clearance

**DOI:** 10.1186/s13054-019-2308-x

**Published:** 2019-01-16

**Authors:** Thibaud Besnard, Cédric Carrié, Laurent Petit, Matthieu Biais

**Affiliations:** 1 Anesthesiology and Critical Care Department, CHU Bordeaux, 33000 Bordeaux, France; 2 Université Bordeaux Segalen, 33000 Bordeaux, France; 3 Surgical and Trauma Intensive Care Unit, Anesthesiology and Critical Care Department, Hôpital Pellegrin, CHU Bordeaux, Place Amélie Raba Léon, 33076 Bordeaux Cedex, France

**Keywords:** Augmented renal clearance, Piperacillin, Critical care

Dear Editor,

In intensive care settings, augmented renal clearance (ARC) is recognized as a leading cause of subtherapeutic antibiotic exposure, and piperacillin-tazobactam (PTZ) has been the most frequently studied antibiotic in this context [1–5] (Table 1). We, like others, previously suggested that higher than licensed dosing regimens should be necessary for empirical treatment in patients with ARC [4, 5]. We thus aimed to determine the efficacy and tolerability of such a strategy.

**Table 1:** Documented rates of pharmacodynamic target non-attainment for various piperacillin dosing regimens in ARC patients

Study	Population	PIP dosing regimen and administration	ARC	Probability of achieving a 100%fT_>16mg/L_ in ARC patients
Andersen et al. [1]	22 non-critically ill patients	4 g every 8 h 3-min bolus	eCl_Cr_ > 130 mL/min *N* = 4/22 (18%)	In ARC patients, probability of achieving a 100%fT_> 16 mg/L_ was 0%.
Udy et al. [2]	48 critically ill patients	4 g/0,5 g every 6 h 20-min intermittent infusion	6-h mCl_Cr_ as continuous variable	Cumulative fraction of response decreased from 40% to less than 5% when Cr_CL_ values increased from 120 to 300 mL/min.
Carlier et al. [3]	60 critically ill patients 43 treated by PIP	4 g/0,5 g every 6 h 3-h extended infusion	24-h mCl_Cr_ > 130 mL/min *N* = 29/60 (48%)	In ARC patients, probability of achieving a 100%fT_> 16 mg/L_ was 24% [*no specific data for PIP*].
Carrié et al. [4]	59 critically ill patients 173 PIP plasma samples	16 g/day continuously 160 mg/mL, 12-h infusion	24-h mCl_Cr_ > 130 mL/min *N* = 36/59 (61%)	Probability of achieving a 100%fT_> 16 mg/L_ was 93% for 130 ≤ Cr_CL_ < 200 mL/min and 80% for Cl_Cr_ > 200 mL/min.
Dhaese et al. [5]	110 critically ill patients 270 PIP plasma samples	Continuous infusion, dosing regimen based on kidney function (16–24 g/day)	8-h mCl_Cr_ > 130 mL/min *N* = 77/270 (32%)	The fractional target attainment for the standard dosing regimen (16 g/day) decreased from 75 to 37% when Cr_CL_ increased from 150 to 300 mL/min.

*%fT*_*> 16 mg/L*_ fraction of time spent with an unbound concentration > 16 mg/L (representing the highest MIC for *Pseudomonas* as per the European Committee on Antimicrobial Susceptibility Testing), *ARC* augmented renal clearance, *eCl*_*Cr*_ estimated creatinine clearance (Cockroft and Gault), *mCl*_*Cr*_ measured creatinine clearance, *FTA* fractional target attainment, *MIC* minimal inhibitory concentration, *PIP* piperacillin

For this purpose, we performed a retrospective analysis of our local database over a 10-month period (February to November 2018). Ethical approval confirmed the observational design of the study (IRB number: CERAR 00010254-2018-074). Over the study period, every patient with a 24-h measured creatinine clearance (CL_Cr_) ≥ 150 mL/min received increased dosing regimens of PTZ (20/2.5 g daily after a loading dose of 4/0.5 g over 60 min) [4]. Subsequent dose adjustments were guided by therapeutic drug monitoring performed between 24 and 72 h of antimicrobial therapy. As previously described, observed concentrations were corrected for protein binding (30% for piperacillin) to estimate unbound fraction [1].

As MIC data are often not available to the clinician prescribing an empirical antimicrobial regimen, piperacillin underdosing was defined by an unbound concentration under 16 mg/L, representing the highest MIC for *Pseudomonas* as per the European Committee on Antimicrobial Susceptibility Testing (EUCAST) [4]. Empirical underdosing for tazobactam was defined by an unbound concentration under 2 mg/L, representing the highest MIC for high-level β-lactamase-producing strains [4]. Excessive dosing was defined as a free drug concentration above 150 mg/L [4].

The final dataset consisted of 36 PTZ samples collected from 35 patients. The main characteristics and outcomes of these patients are resumed in Table 2. Except for one tazobactam sample, all samples were in the therapeutic range (Fig. 1). No patient experienced excessive dosing above the supposed toxic cutoff ≥ 150 mg/L. Three of them (9%) experienced therapeutic failure or relapse [4], all related to secondary acquisition of antimicrobial resistance.

**Table 2:** Characteristics of the population

Variable	Overall population *N* = 35
Demographic data	
- Age (years)	48 [37–57]
- Male sex	31 (89)
- BMI (kg/m^2^)	25 [22–29]
Admission	
- Polytrauma	30 (86)
- Non-traumatic surgery	5 (14)
SAPS II	42 [34–51]
Presumed/confirmed site of infection	
- Pulmonary infection	31 (89)
- Intra-abdominal infection	3 (9)
- Intravascular-catheter-related infection	1 (3)
Bacteremia	2 (6)
Use of vasopressors	12 (34)
Modified SOFA score*	3 [1–6]
CL_Cr_ the day of therapeutic drug monitoring	166 [159–191]
Antimicrobial therapy	
- Duration of antibiotic therapy before TDM	2 [1–3]
- Association with aminoglycoside or quinolone	8 (23)
- De-escalation	9 (26)
- Total duration of antimicrobial therapy (days)	7 [5–7]
Type of pathogen	
- Enterobacteriaceae	33 (94)
- *Staphylococcus* spp.	18 (51)
- *Haemophilus influenzae*	8 (23)
- Non-fermenting GNB	3 (9)
- Other	3 (9)
Polymicrobial infection	20 (57)
Non-documented infection	1 (3)
PK/PD targets	
- Piperacillin unbound concentrations (mg/L)	36.4 [27.7–44.3]
Empirical underdosing for piperacillin	0 (0)
Excessive dosing for piperacillin	0 (0)
- Tazobactam unbound concentrations (mg/L)	4.55 [3.57–5.88]
Empirical underdosing for tazobactam	1 (3)
- PIP/TAZ ratio	9.1 [6.9–11.1]
Clinical outcomes	
- Therapeutic failure before end of treatment	2 (6)
- Relapse after end of treatment	1 (3)
Secondary resistance to PTZ	3 (9)
MV duration (days)	14 [4–26]
ICU length of stay (days)	22 [14–37]
ICU mortality	0 (0)

Results expressed as median [25–75 interquartile] and numbers (percentage). Therapeutic failure was defined as an impaired response (persistent or recurrent fever, organ dysfunction, clinical and biological symptoms of the initial infection) with the need for escalating empirical antimicrobial therapy. Relapse was defined by a recurrent infection within 15 days after completing antibiotic therapy with at least one of the initial causative bacterial strains growing at a significant concentration from a second sample

*Sepsis-related Organ Failure Assessment score, without neurologic and renal components

**Fig. 1:**
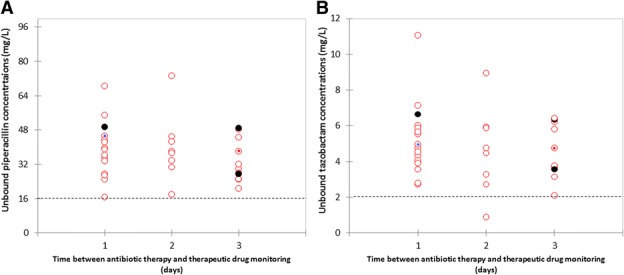
Unbound steady-state concentrations (mg/L) of piperacillin (**a**) and tazobactam (**b**) using higher than licensed dosing regimens (20 g/day [160 mg/mL, 10-h infusion] after a loading dose of 4 g) in critically ill patients with ARC (Cl_Cr_ ≥ 150 mL/min the first day of antimicrobial therapy). The dotted line indicates underdosing threshold for piperacillin (fixed at 16 mg/L) and tazobactam (fixed at 2 mg/L) [4]*.* The black circles indicate samples from patients who experienced therapeutic failure [4]

When targeting a theoretical MIC at the upper limit of the susceptibility range, higher than licensed doses of PTZ allowed achieving the pharmacodynamic target in all patients with CL_Cr_ ≥ 150 mL/min, without excessive dosing. Further studies are warranted to confirm if such a strategy improves the rate of therapeutic success.

## Abbreviations

*%fT*_> 16 mg/L_: Fraction of time spent with an unbound concentration > 16 mg/L (representing the highest MIC for *Pseudomonas* as per the European Committee on Antimicrobial Susceptibility Testing)
ARC: Augmented renal clearance
Cl_Cr_: Creatinine clearance
eCl_Cr_: Estimated creatinine clearance (Cockcroft and Gault)
FTA: Fractional target attainment
mCl_Cr_: Measured creatinine clearance
MIC: Minimum inhibitory concentration
PIP: Piperacillin
PTZ: Piperacillin-Tazobactam

## References

[1] Andersen MG, Thorsted A, Storgaard M, Kristoffersson AN, Friberg LE, Öbrink-Hansen K. Population pharmacokinetics of piperacillin in sepsis patients: should alternative dosing strategies be considered? Antimicrob Agents Chemother. 2018;62(5). 10.1128/AAC.02306-17 PMC5923116 29507062

[2] Udy AA, Lipman J, Jarrett P, Klein K, Wallis SC, Patel K, Kirkpatrick CM, Kruger PS, Paterson DL, Roberts MS, Roberts JA (2015). Are standard doses of piperacillin sufficient for critically ill patients with augmented creatinine clearance?. Crit Care.

[3] Carlier M, Carrette S, Roberts JA, Stove V, Verstraete A, Hoste E, Depuydt P, Decruyenaere J, Lipman J, Wallis SC, De Waele JJ (2013). Meropenem and piperacillin/tazobactam prescribing in critically ill patients: does augmented renal clearance affect pharmacokinetic/pharmacodynamic target attainment when extended infusions are used?. Crit Care.

[4] Carrié C, Legeron R, Petit L, Ollivier J, Cottenceau V, d'Houdain N, Boyer P, Lafitte M, Xuereb F, Sztark F, Breilh D, Biais M (2018). Higher than standard dosing regimen are needed to achieve optimal antibiotic exposure in critically ill patients with augmented renal clearance receiving piperacillin-tazobactam administered by continuous infusion. J Crit Care.

[5] Dhaese SAM, Roberts JA, Carlier M, Verstraete AG, Stove V, De Waele JJ (2018). Population pharmacokinetics of continuous infusion of piperacillin in critically ill patients. Int J Antimicrob Agents.

